# IMPROVING MOOD THROUGH PHYSICAL ACTIVITY FOR CARERS AND CARE RECIPIENTS TRIAL (IMPACCT): RESULTS OF A RCT

**DOI:** 10.1093/geroni/igz038.588

**Published:** 2019-11-08

**Authors:** Briony Dow, Susan (Sue) Malta, Ellen Gaffy, Melissa Russell, Sue Williams, Kirsten J Moore, Nicola Lautenschlager, Samantha Loi

**Affiliations:** 1 National Ageing Research Institute, Parkville, Australia; 2 University of Melbourne, Victoria, Australia; 3 University of Melbourne, Parkville, Australia; 4 University College London, London, United Kingdom

## Abstract

The aim of this Australian study was to investigate effects on depression of a 6-month individually tailored home-based exercise program for caregivers, designed to be done with the person they care for. Ninety-one caregiver-care recipient dyads and 30 caregiver-only participants (caregivers scoring ≥4 on the 15 item Geriatric Depression Scale (GDS-15)) were randomized into one of three groups: exercise intervention (n=50, 34 dyads and 16 caregiver only), social support control (n= 50, 42 dyads and 8 caregiver only) or usual care control (n= 21, 15 dyads and 6 caregiver only). The exercise group completed an individualised program based on the Otago-plus. The primary outcome was the proportion of participants with GDS-15 ≤4. Outcome assessors were blinded to group assignment. There were no significant difference in depression between the physical activity intervention group and the social control (OR 1.06, 95% Confidence Interval (CI) 0.44, 2.56) and the physical activity intervention group and the usual care control (OR 1.51 95% CI 0.46, 4.94) at six months or at 12-months. However, more than 50% of caregivers in all three groups no longer had a GDS-15 score >4 at 6 months. Sub-group analysis revealed that after 6 months caregivers in the exercise group caring for someone with an MMSE ≥24 were significantly less depressed than those caring for someone with an MMSE score of <24 compared with social (p value <0.02) and usual care groups (p value < 0.02). A dyad exercise intervention may be beneficial for those caring for someone without cognitive decline.

